# The Linkage Between Ecotoxins Within Maximum Permissible Concentrations, Oxidative Stress and Antibodies Against Cyclic Citrullinated Peptides in Patients and Persons at Preclinic Stages of Rheumatoid Arthritis

**DOI:** 10.3390/ijms27073328

**Published:** 2026-04-07

**Authors:** Igor L. Serdiuk, Anna R. Valeeva, Sergei V. Petrov, Damir G. Salikhov, Gevorg G. Kazarian, Marina O. Korovina, Olga A. Kravtsova, Elena I. Shagimardanova, Wesley Brooks, Oleg R. Badrutdinov, Malik N. Mukminov, Eduard A. Shuralev, Nikolai D. Shamaev, Andrej A. Novikov, Yves Renaudineau, Marina I. Arleevskaya

**Affiliations:** 1 Central Research Laboratory, KSMA–Branch Campus of the FSBEI FPE RMACPE MOH, Butlerov St, 36., Kazan 420012, Tatarstan Republic, Russia; dr.serdyuc@mail.ru (I.L.S.); anna-valeeva@mail.ru (A.R.V.); sergeipetrov96@yandex.ru (S.V.P.); gevorg.kazarian@mail.ru (G.G.K.); koporulina.mo@gmail.com (M.O.K.); malik-bee@mail.ru (M.N.M.); eduard.shuralev@mail.ru (E.A.S.); nikolai.shamaev94@mail.ru (N.D.S.); 2 Institute of Fundamental Medicine and Biology, Kazan Federal University, Kremlyovskaya Str, 18, Kazan 420008, Tatarstan Republic, Russia; 3 Institute of Ecology, Biotechnology and Nature Management, Kazan Federal University, Kremlyovskaya Str, 18, Kazan 420008, Tatarstan Republic, Russia; sdamirv5@yandex.ru (D.G.S.); obadrut@mail.ru (O.R.B.); 4 Genomics and Bio-Imaging Core Facility, Moscow 121205, Russia; 5 Life Improvement by Future Technologies (LIFT) Center, Moscow 121205, Russia; 6 Department of Chemistry, University of South Florida, Tampa, FL 33620, USA; 7 Novikov Laboratories LLC, Office 202, St. Krasnokokshayskaya 189a, Kazan 420033, Tatarstan Republic, Russia; andrei@hobukob.ru; 8 Referral Medical Biology Laboratory, Department of Immunology, Toulouse University Hospital Center, University of Toulouse, 330 Avenue de Grande-Bretagne, 31000 Toulouse, France; renaudineau.y@chu-toulouse.fr

**Keywords:** rheumatoid arthritis, preclinic, atmospheric pollutants, soil heavy metals, oxidative stress, antibodies to cyclic citrullinated peptide

## Abstract

Environmental factors are suspected of triggering rheumatoid arthritis (RA). One such factor is oxidative stress (OS), which is a step in ecotoxin detoxification and a damaging factor. The linkage of ecotoxin-triggered OS with clinical and laboratory RA indices in patients and individuals at the pre-RA stage was studied in patients with early (e) (n = 35) and advanced (a) stages of RA (n = 25) and individuals at pre-RA stages (FDR-First-Degree Relative(s), pre-RA, n = 72) in comparison with 52 healthy individuals without autoimmune and immunoinflammatory diseases in their family history (Controls). Ecotoxins within permissible concentration limits were associated with serum levels of OS markers in all cohorts, including Controls. Serum oxidized low-density lipoprotein (oxLDL) levels in pre-RA and eRA cohorts exceeded Control values. Significant differences were found in anti-oxLDL antibody correlations and OS markers. In pre-RA and both RA cohorts, a relationship was found with regards to serum antibodies to cyclic citrullinated peptide (ACCP) levels. Thus, ecotoxin-induced OS likely triggers pathogenic mechanisms at the pre-RA stage and RA onset.

## 1. Introduction

Rheumatoid arthritis (RA) is a recognized model of multifactorial diseases developing as an inappropriate response to environmental challenges. The list of alleged environmental triggers includes ecotoxins [1]. An increased risk of RA developing in connection with exposure to certain ecotoxins was demonstrated [2,3,4], while in model experiments, a role in RA pathogenesis was revealed for ecotoxin interference with key processes [5,6,7,8]. In particular, one of the basic processes triggered by environmental factors is oxidative stress (OS) [9,10,11].

The syndrome of nonspecific increased chemical susceptibility due to the individual dispersive efficacy of ecotoxin degradation mechanisms was described [12]. RA-associated SNPs in detoxication system enzymes were revealed [13,14]. So, the assumption seems quite reasonable that there is a possible trigger effect of ecotoxins in individuals at risk for RA that is more pronounced than in the general population.

We aimed to study the impact of ecotoxins within maximum permissible concentrations (MPC) on the development of OS and ecotoxin-triggered OS links with clinical and laboratory RA indices in the cohorts of residents of administrative districts of the Republic of Tatarstan (RT): early (e) and advanced (a) RA patients, individuals at the preclinical stage (pre-RA) and conditionally healthy individuals without autoimmune and immunoinflammatory diseases in their family history (Controls).

## 2. Results

### 2.1. The Link Between Certain Environmental Factors and Serum Markers of Oxidative Stress

**Figure 1:**
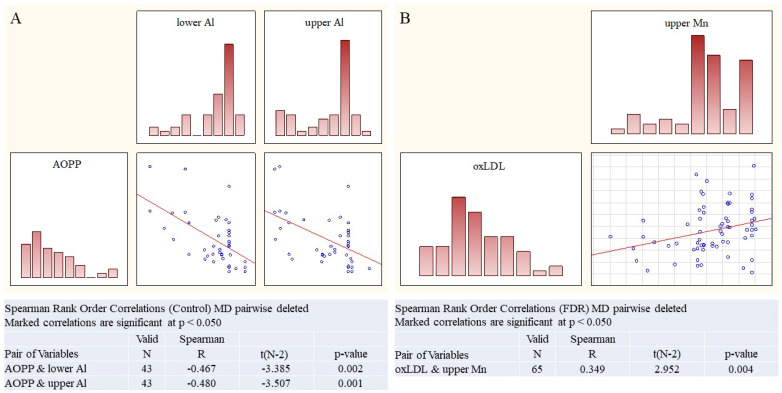
The link between environmental factors and serum markers of oxidative stress in control and pre-RA cohorts. **Control cohort**: (**A**) AOPP and Al concentration in lower and upper soil layers; **pre-RA cohort:** (**B**) oxLDL and Mn concentration in the upper soil layer. Spearman rank correlation, R, *p*.

In **Controls** (Figure 1A), a weak but highly reliable correlation was found between AOPP (advanced oxidation protein products) levels and Al in the lower (R = −0.47, *p* = 0.002) and upper (R = −0.48, *p* = 0.001) soil layers.

In the **pre-RA cohort** (Figure 1B, a highly reliable weak correlation of serum oxLDL levels and Mn concentration in the upper soil layer was demonstrated (R = 0.35, *p* = 0.004).

**Figure 2:**
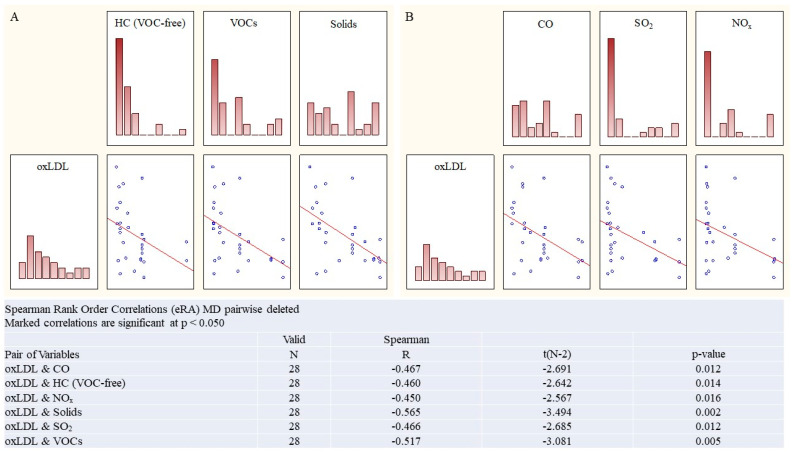
The link between environmental factors and serum markers of oxidative stress in eRA and aRA cohorts. Group eRA: (**A**) oxLDL, carbon monoxide and VOCs; Group aRA: (**B**) AT-oxLDL and sulfur oxide; Spearman’s rank correlation, R, *p*.

In the **eRA cohort** (Figure 2), serum oxLDL levels correlated with concentrations of all the monitored indicators of atmospheric ecotoxin concentrations: carbon monoxide (CO) (R = 0.47, *p* = 0.01), VOC-free hydrocarbons (HC) (R = 0.47, *p* = 0.01), nitrogen oxides (NO_x_) (R = 0.45, *p* = 0.02), solids (R = 0.57, *p* = 0.002), sulfur dioxide (SO_2_) (R = 0.44, *p* = 0.01), and volatile organic compounds (VOCs) (R = 0.51, *p* = 0.005).

**Figure 3:**
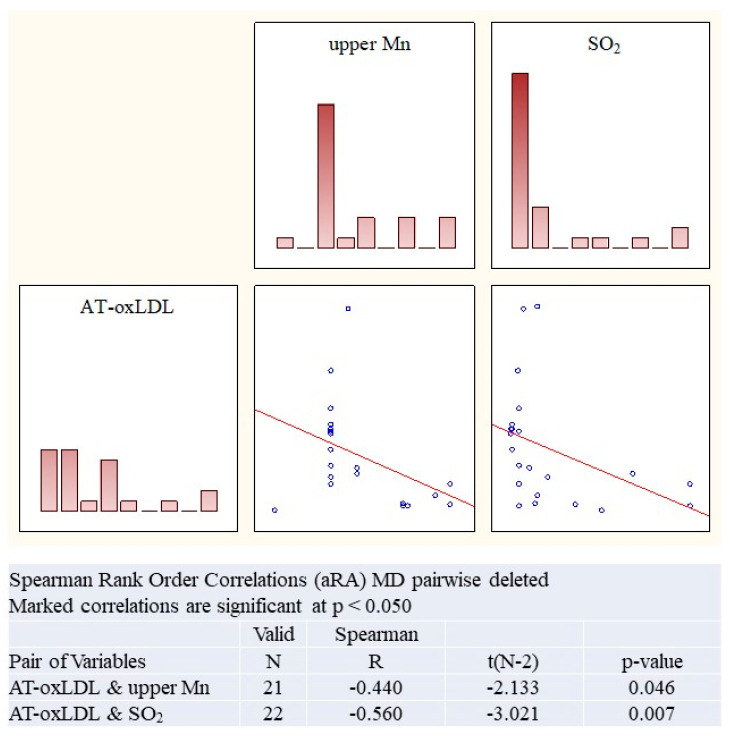
The link between environmental factors and serum markers of oxidative stress in the aRA cohorts. AT-oxLDL and sulfur oxide; AT-oxLDL and Mn in the upper soil layer. Spearman’s rank correlation, R, *p*.

In the **aRA cohort** (Figure 3), serum AT-oxLDL (anti-oxidized low-density lipoprotein antibody) levels correlated with atmospheric sulfur dioxide (R = 0.56, *p* = 0.007) and, presumably, the upper soil layer Mn (R = 0.44, *p* = 0.05).

So, the link between OS markers and certain ecotoxins was traced in all the cohorts, including Controls.

### 2.2. Complex Impact of Ecotoxin Combinations on Oxidative Stress Markers

Multiple regression analysis demonstrated that the complex impact of ecotoxin combinations makes a significant contribution to the provocation of OS in **Controls** (Supplementary S1). In the models, including certain atmospheric ecotoxins and soil heavy metals, the 8-OHdG (8-hydroxy-2′-deoxyguanosine) data spread was reduced by 80% (*p* < 0.01148), AOPP data spread was reduced by 87% (*p* < 0.00739), oxLDL data spread was reduced by 73% (*p* < 0.01236), and AT-oxLDL level data spread was reduced by 74%, with presumable significance (*p* < 0.02534).

Analysis of the results in both RA cohorts demonstrated that the various combinations of ecotoxins made an evident contribution to the development of OS (Supplementary S1).

In the **eRA** cohort: in the models, including certain atmospheric ecotoxins and the soil heavy metals, the 8-OHdG data spread was reduced by 99% (*p* < 0.00000), AOPP data spread was reduced by 87% (*p* < 0.00724), oxLDL data spread was reduced by 77%, with presumable significance (*p* < 0.04444), and AT-oxLDL level data spread was reduced by 82%, with presumable significance (*p* < 0.05231).

In the **aRA** cohort: the 8-OHdG data spread was reduced by 97% (*p* < 0.00001), AOPP data spread was reduced by 94% (*p* < 0.00004), oxLDL data spread was reduced by 71%, with presumable significance (*p* < 0.02505), and AT-oxLDL level data spread was reduced by 92% (*p* < 0.00531).

In the **FDR cohort:** the AOPP data spread was reduced by 42%, with presumable significance (*p* < 0.02353), oxLDL data spread was reduced by 65% (*p* < 0.00980), and AT-oxLDL level data spread was reduced by 49%, with presumable significance (*p* < 0.02688). We failed to find combinations of ecofactors reliably associated with 8-OHdG levels in this cohort (Supplementary S1).

Thus, the effect of ecotoxin combinations on oxLDL levels was similar in all cohorts, including Controls. The impacts of ecotoxin combinations on AOPP levels and the presumable impact on AT-oxLDL production in the Control and both RA cohorts were also comparable. The impact of ecotoxin combinations on 8-OHdG levels might have been more pronounced in both RA cohorts compared to Controls, without raising objections, since increased sensitivity to xenobiotic exposure and ROS-induced DNA damage in RA was demonstrated [15,16].

The results of the multiple regression analysis in the FDR cohort were paradoxical. One might expect the FDR cohort to show similar impacts of environmental toxicants on OS markers to those in Controls, given their possible lack of influence on OS at the pre-RA stage, or effects similar to those in RA, given the same disturbances in detoxification mechanisms. However, the impact of ecotoxins on AOPP and AT-oxLDL levels was lower than in RA and Controls, and no effect on 8-OHdG was revealed at all. Adding age into the models was ineffective (results not shown). It should be noted that the FDR cohort included individuals at all pre-RA stages from 1st to 4th, which might have influenced the results of the analysis.

### 2.3. Comparison of Serum Markers of Oxidative Stress in Cohorts

**Figure 4:**
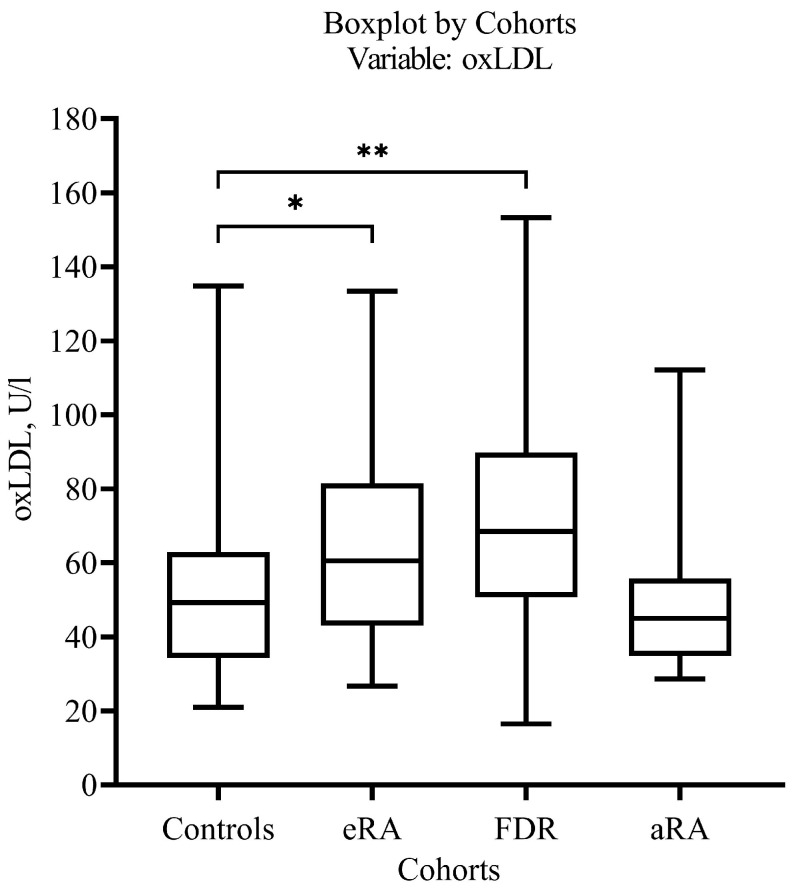
Serum levels of oxLDL in the eRA, aRA, pre-RA and Control cohorts. Mann–Whitney U Test, * *p* = 0.024, ** *p* = 0.001.

The levels of oxLDL were higher in the pre-RA (*p* = 0.001399) and eRA (*p* = 0.024375, presumable significance) cohorts vs. Controls (Table 1, Figure 4).

AT-oxLDL levels were higher in the aRA cohort vs. Controls (*p* = 0.022769, presumable significance).

### 2.4. The Link Between Serum Levels of OS Markers and At-oxLDL in Cohorts

Spearman correlation analysis revealed interesting differences in the associations between AT-oxLDL levels and certain OS markers in the cohorts (Table 2).

In **Control**s, AT-oxLDL levels correlated with all the OS markers: 8-OHdG (R = −0.507879, *p* = 0.001134), oxLDL (R = −0.302626, *p* = 0.054456, presumable significance), and AOPP (R = 0.577613, *p* = 0.000280). The AT-oxLDL link with oxLDL demonstrated a weaker correlation coefficient than that for the AT-oxLDL links with AOPP and 8-OHdG.

In **FDR**s, AT-oxLDL levels were in a highly reliable correlation with -OHdG (R = −0.353567, *p* = 0.006477), with a weaker correlation coefficient than that in Controls, oxLDL (R = −0.580879, *p* = 0.000001), and AOPP (R = 0.666826, *p* = 0.000000).

In the **eRA** cohort, the links of AT-oxLDL with oxLDL (R = −0.537796, *p* = 0.005558) and with AOPP (R = 0.609395, *p* = 0.001572) levels were revealed, while the link with 8-OHdG was lost. In the **aRA** cohort, no AT-oxLDL and OS marker correlations were revealed (Supplementary S2).

Purely hypothetical, this could be due to the low affinity of AT-oxLDL with a priori sanitary function and greater affinity in the FDR and eRA cohorts. With regard to aRA, the loss of correlation between the levels of AT-oxLDL and other OCs, including oxLDL, might indicate their uncontrolled synthesis. Of course, this is nothing more than a guess that requires further research (affinity assays, epitope mapping).

### 2.5. The Link of Serum Levels of OS Markers and Accp Levels in Cohorts

Serum ACCP levels appeared as a single RA index linked with ecotoxins and OS marker levels.

In the **Control** cohort, based on the ACCP criteria, the levels had a narrow range of normal values (inclusion criteria).

In the **FDR** cohort, a combination of OS markers influenced ACCP levels—in the multiple regression model data, the spread was reduced by 58% (*p* < 0.00016)—and ecotoxin combinations—the data spread was reduced by 71%, with presumable significance (*p* < 0.01983) (Supplementary S3).

**Figure 5:**
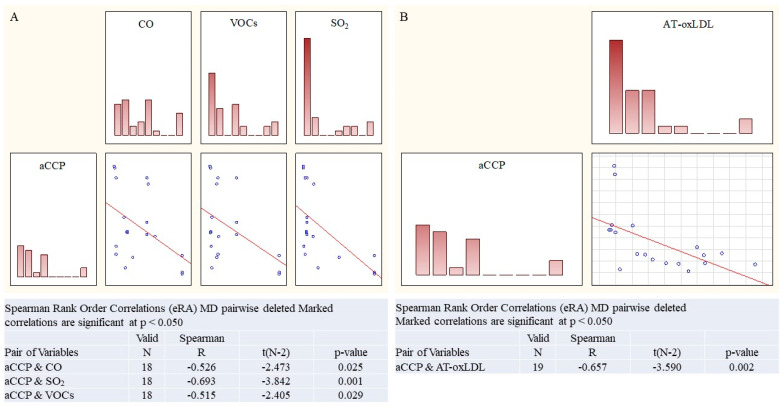
Correlation of ACCP with ecotoxin and AT-oxLDL levels in eRA cohort. (**A**) ACCP and ecotoxins; (**B**) ACCP and AT-ox-LDL. Spearman’s rank correlation, R, *p*.

In the **eRA** cohort, multiple regression analysis revealed the impact on ACCP levels of OS marker combinations—the data spread was reduced by 62%, with presumable significance (*p* < 0.03163)—and of ecotoxin combinations—the data spread was reduced by 94% (*p* < 0.00340) (Supplementary S3). More than that, ACCP levels were in the reliable Spearman correlations with carbon monoxide (R = 0.525909, *p* = 0.024979), volatile organic compounds (VOCs) (R = 0.515314, *p* = 0.028622), and sulfur dioxide (R = 0.692749, *p* = 0.001438); AT-oxLDL was the only OS marker that correlated with ACCP (R = 0.656717, *p* = 0.002255) (Figure 5).

In the **aRA** cohort, a combination of OS markers was linked with ACCP levels—in the multiple regression model, the data spread was reduced by 94% (*p* < 0.00911) (Supplementary S3). No pairwise Spearman correlations of ACCP and OS markers or ecotoxins were revealed. We failed to find any ecotoxin combinations that might be linked with the ACCP level in multiple regression testing.

### 2.6. The Link of Serum Levels of OS Markers in HLADRB1 Shared Epitope Carriers

The link of HLADRB1 shared epitopes (SE) and serum levels of OS markers, on one hand, and SE impact on the relationship of OS markers with RA indices, on the other hand, was analyzed in a combined cohort of Control, FDR, and RA patients. No difference in OS marker levels was revealed in the SE-carriers and non-carriers (Mann–Whitney U Test, results are not shown).

However, only in SE non-carriers, a contribution of OS markers to ACCP levels was detected. In a multivariate regression model including 8-OHdG and AT-oxLDL, the data dispersion decreased by 59% (R = 0.58543218, *p* < 0.00802), whereas in SE-carriers, no links of OS markers and ACCP levels were revealed (Supplementary S4).

### 2.7. The Link of Serum Levels of OS Markers and Some Physiological and Lifestyle–Related Factors

**Figure 6:**
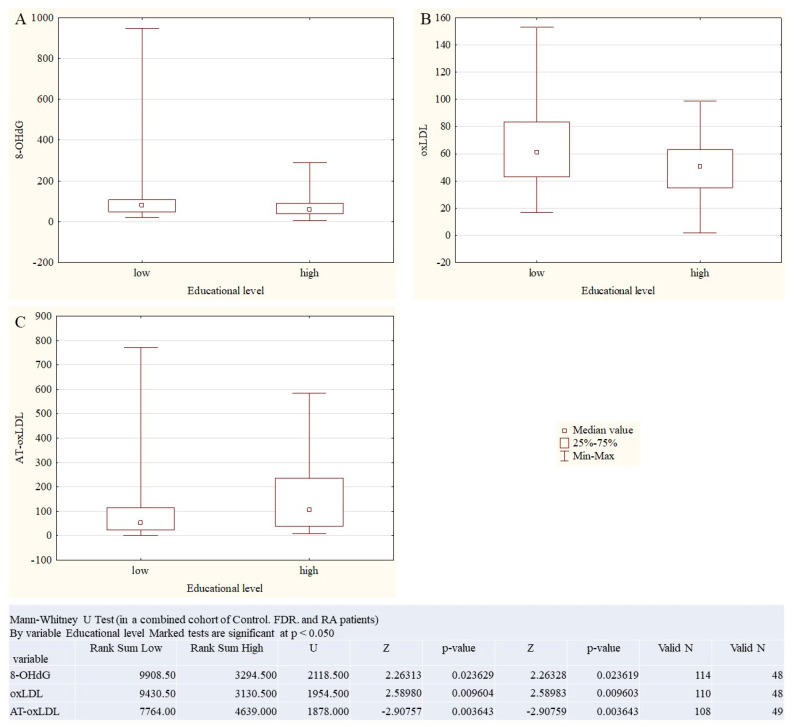
The link of educational level and OS marker concentrations in a combined cohort of Control (**A**), FDR (**B**), and RA (**C**) patients. Mann–Whitney U Test.

The link of educational level and OS marker concentrations was revealed in a combined cohort of Control, FDR, and RA patients (Mann–Whitney U Test, Figure 6), For statistical purposes, education was dichotomized into low educational level (secondary and high school graduates, n = 123) and high educational level (university graduates, n = 53).

Higher educated persons had lower oxLDL (*p* = 0.009603), 8-OHdG levels (*p* = 0.023619) and higher AT-oxLDL (*p* = 0.02) vs. those with a lower educational level. However, in the highly educated cohort, the average age and BMI were significantly lower than in the low educational level cohort (Table 3).

Age and BMI were in a reliable direct correlation with each other, and each of them significantly correlated with the levels of oxLDL (direct relationship) and with AT-oxLDL (inverse relationship) (Table 3 and Table 4).

However multiple regression analysis revealed a combined effect of age and BMI only on oxLDL levels, and it was rather weak yet reliable—in the model including these two factors, the data spread was reduced by 26% (*p* < 0.00566).

We found no differences in OS marker levels from fish and coffee consumption in the combined cohort of controls, FDRs and RA patients (Mann–Whitney U Test, data not presented). It turned out that coffee consumption was limited to 1–2 cups per day, so the dose-dependent effect was not analyzed.

Individuals in our cohorts either abstained from alcohol or consumed it infrequently. Only a few reported regular alcohol consumptions. No differences in OS marker levels were found between no and infrequent alcohol consumption.

Active tobacco smokers were also rare. Therefore, we analyzed the differences of OS marker levels between individuals with no exposure to tobacco and active and/or passive smokers. No differences in the levels of OS markers were found as well.

No correlations of number of births in women and OS marker levels were revealed (Spearman rank order correlations. data not shown).

It is possible that the relationship between OS marker levels and education level is not determined by any single factor but rather by a combination of lifestyle factors (including income level. health concerns) as well as the nature of one’s professional activities. At least, our analysis of the eating habits we tested at the yes/no level revealed certain differences when comparing individuals with higher and lower educational levels (Table 5).

The contribution of ecotoxins and physiological/lifestyle-related factors was analyzed using the multiple regression method separately in SE-carriers and non-carriers (Supplementary S5).

A significant contribution of the ecotoxin combinations to 8-OHdG levels was revealed both in SE-carriers and non-carriers—the data spread was reduced by more than 90%, with high reliability. The inclusion of age, BMI and numbers of births did not change the results, and in the models including only these factors, no links with 8-OHdG levels were revealed.

In SE-carriers. a strong link of oxLDL levels and a set of ecotoxins was found—the data spread was reduced by 91% (*p* < 0.00952). The influence of age, BMI, and number of births on this marker was also significant, although not as pronounced as the association with ecotoxins—the data spread was reduced by 52% (*p* < 0.00715). In non-carriers, a link of oxLDL levels and a set of ecotoxins was also found, yet it looked to be weaker—the data spread was reduced by 76%, with presumable significance (*p* < 0.02038). Adding age, BMI, and number of births to the model in addition to the total ecotoxins, as well as the model that included only these three individual factors, did not reveal a link with the indicated three individual factors (Supplementary S5).

AOPP levels were influenced by a combination of ecotoxins, regardless of SE status, with presumable significance. Adding age, BMI, and number of births to the ecotoxins made the model more significant only in non-carriers of SE.

In SE-carriers, no effect of the combination of ecotoxins on AT-oxLDL levels was found. When age, BMI, and number of births were added to this model, the data spread was reduced by 76%, with presumable significance (*p* < 0.02714). The model including the indicated three factors revealed no effect on AT-oxLDL levels in SE-carriers. In contrast to SE-carriers. a highly significant effect on AT-LDL levels was found in non-carriers in a model including only ecotoxins—the data spread was reduced by 92% with presumable significance (*p* < 0.00705). Including individual factors in this model somewhat worsened its reliability (*p* < 0.01495).

So. some differences in the contributions of ecotoxins and individual factors to OS marker levels were identified in SE-carriers and non-carriers. Further research into this issue is clearly needed.

### 2.8. The Link of Serum Levels of OS Markers, ACCP Levels and Ecotoxins at the Pre-Clinical RA Stages

No trends in differences in the levels of OS markers at pre-RA stages 1–4 and in eRA were detected (Kruskal–Wallis and Mann–Whitney methods. data not shown).

However. a comparison of pre-RA stages and eRA using the Kruskal–Wallis method allowed us to identify the following ecotoxins that were significant for progression through the stages (Supplementary S6):

Atmospheric ecotoxins: CO (*p* = 0.0054), SO2 (*p* = 0.0460), VOCs (*p* = 0.0125), solids (*p* = 0.0140).

In upper soil layer: Zn (*p* = 0.0031), Pb (*p* = 0.0183), Mn (*p* = 0.0138), Cu (*p* = 0.0311), As (*p* = 0.0209), Al (*p* = 0.0054), Se (*p* = 0.0074).

In lower soil layer: Zn (*p* = 0.0050), Pb (*p* = 0.0476), Mn (*p* = 0.0040), Cu (*p* = 0.0422), Co (*p* = 0.0268), Sr (*p* = 0.0052).

The next step was to analyze the link of ecotoxin levels and pre-RA stages using the Mann–Whitney method. Next, we compiled a list of ecotoxins with significantly different levels at pre-RA stages in a pairwise comparison between stages (Mann–Whitney test) (Supplementary S7).

The following ecotoxins might be important for evolution through pre-RA stages (highlighted in yellow in Supplementary S7):

Atmospheric ecotoxins: SO2, VOCs, solids, CO.

In upper soil layer: Co, Cu, Mn, Pb, Se, Al, Zn, As, Cd, Mo.

In lower soil layer: Co, Cu, Mn, Pb, Se, Sr, Zn.

Next. we attempted to analyze whether ecotoxins contributed to the OS level in pre-RA stages. Given the relatively small sample sizes and the set of ecotoxins presumably significant for evolution through the pre-RA stages, multiple regression analysis was conducted in a pooled cohort of individuals at pre-RA stages 2–4 (Supplementary S8). Atmospheric ecotoxins and soil heavy metals in various combinations had an effect on 8-OHdG levels (reducing data scatter by 53%. *p* < 0.03366), oxLDL levels (reducing data scatter by 54%. *p* < 0.03645) and AT-oxLDL levels (reducing data scatter by 63%. *p* < 0.00921). In the pre-RA stage 3 cohort. sets of ecotoxins had an effect on 8-OHdG levels (reducing data scatter by 71%. *p* < 0.03582), oxLDL levels (reducing data scatter by 91%. *p* < 0.01283), and AT-oxLDL levels (reducing data scatter by 83%. *p* < 0.00995). In a smaller pre-RA stage 4 cohort, the impact of the sets of ecotoxins on 8-OHdG was on the verge of significance (*p* < 0.05557), yet the effect on oxLDL levels was demonstrated (reducing data scatter by 91%. *p* < 0.01283), and for AT-oxLDL levels (reducing data scatter by 83%. *p* < 0.03448), the spread of AT-oxLDL values was reliably reduced by more than 90% in two tested models.

The link of ACCP levels and OS markers was analyzed in the following cohorts of persons at pre-RA stages (Supplementary S9):

- Combined cohort of persons at 2–4 pre-RA stages;
- Combined cohort of persons at 3–4 pre-RA stages;
- Persons at 3 pre-RA stage;
- Persons at 4 pre-RA stage.

In the listed cohorts. multiple regression analysis revealed the link of ACCP serum levels and a set of OS markers:

At stages 2–4. data scatter was reduced by 58% (*p* < 0.00016);

At stages 3–4. data scatter was reduced by 48% in the model not including AT-oxLDL. with presumable significance (*p* < 0.05088);

At stage 3. data scatter was reduced by 95% (*p* < 0.00000);

At stage 4. data scatter was reduced by 82%. with presumable significance (*p* < 0.04529).

## 3. Discussion

Since rheumatoid arthritis is a multifactorial immune–inflammatory disease that develops as a result of an inadequate response of a genetically predisposed organism to external challenges, interest in the role of environmental factors in provoking the disease is natural.

In some publications. an increased risk of RA development was demonstrated in connection with contact with some ecotoxins [2,3,4].

It is fueled by other sets of research findings.

The experiments on animal models and cell cultures demonstrated the interference of ecotoxins in basic processes. playing important roles in the development of RA. For example. if we list the factors included in our study. heavy metals (Cd, Ni, Co, Cr, Zn) and organic molecules (nitrogen oxide, carbon oxide, hydrocarbons) stimulated the NFkB-signaling pathway. with the following proinflammatory effects [5,17,18,19,20,21].

Another locus *minoris resistencia* in RA is a DNA damage repair disturbance leading to chronic. tissue-damaging inflammation [22]. The heavy metals (Cd, Ni, Cr, Zn) were demonstrated to aggravate these violations [23,24,25,26].

Heavy metals (Cd, Ni, Co, Cr, Cu, Zn, As) and organic molecules (nitrogen oxide, hydrocarbons) provoked OS in model experiments [5,8,9,27].

Another bulk of data indicates a number of RA-associated SNPs of genes encoding factors of the detoxification system: deletion polymorphism of glutathione S transferase—enzyme of phase 2 detoxification of xenobiotics (GSTM1) increased RA susceptibility. particularly non-HLA-DRB1 SE [13]. Allele C3435T enzyme MDR1 3435—transporter for many drugs. xenobiotics influence on the RA activity response to therapy [14]. N acetyltransferase 2 SNPs, involved in the metabolism of many xenobiotics. are due to slow acetylation NAT2. which is a risk factor for joint destruction.

In addition, intriguing results from studies of aryl hydrocarbon receptor (AhR)—transcription factor-mediating xenobiotic effects of many pollutants (including tobacco) in a mouse model were obtained [28]. That researcher revealed a nuclear factor kappaB-mediated synergistic interaction between the SE and AhR pathways. facilitating differentiation of Th17 cells and osteoclasts in severe arthritis. Together with the data that SNPs of genes encoding some enzymes of the detoxification system were associated with non-HLA-DRB1 SE-carriers [13]. It might be reasonable to compare environmental influence in cohorts of HLA-DRB1 SE-carriers and non-carriers.

These data allow us to suspect the so called “syndrome of nonspecific increased chemical susceptibility” [12], if not in everyone, then at least in some individuals predisposed to RA.

We aimed to study OS as a target of ecotoxins, which, on the one hand, plays the role of a damaging factor in RA pathogenesis and. on the other hand, is one of the stages of detoxification.

It should be emphasized that, according to reports from the Ministry of Ecology of RT, emissions of ecotoxins in excess of maximum permissible concentrations (MPC) were not registered in the administrative regions over the analyzed 10 years. The soil heavy metal content also did not exceed MPC.

The results of the statistical analysis demonstrated an undeniable relationship between the levels of OS markers and ecotoxins in all cohorts, including the Controls. It was not so much the influence of a specific ecotoxin that was significant but rather the combination of factors.

As all the individuals included in the analysis were exposed to environmental factors within MPC, which were the same for all residents of the area. the differences in OS parameters that we expected could be attributed to an inadequate response of a predisposed individual to trivial environmental influences in accordance with the concepts of RA.

However. the differences across cohorts were not as pronounced as expected. A comparison of the levels of serum OS markers in the cohorts revealed only an excess of oxLDL concentrations in the FDR and eRA cohorts vs. Controls and excess AT-oxLDL levels in aRA vs. eRA.

It is possible that lipoproteins are most vulnerable already at the pre-RA stage. In the pre-RA cohort of ACCP/RF-positive persons at the arthralgia stage, a lipid disbalance was demonstrated, with worsening as RA progresses from preclinical stages to the onset of RA. associated with a gradually growing inflammatory process [29,30]. The signs of OS in pre-RA persons were revealed as well [31]. It cannot be ruled out that the triggering of OS by ecotoxins is indirect through their aggravation of the frequency and duration of trivial infections to which individuals at the pre-RA stage are susceptible [32,33]. Earlier, we revealed the association of proatherogenic shifts in serum lipid composition with an infectious syndrome in this cohort [30]. It should be noted that some of these infections were due to the increased ROS production by granulocytes [34] and potentially might cause OS.

Analysis of the sets of persons at the preclinical stages 1 (genetic) to 4 (undifferentiated arthritis) and eRA patients demonstrated the role of certain ecotoxins in the evolution of a predisposed individual through pre-RA stages to the disease. The sets of ecotoxins contributed to OS marker levels in the absence of differences in the levels of these indicators at these stages. So, it looks like the most affected in ecotoxin-associated OS was LDL. It should be noted that the various events of ecotoxin interference with lipid metabolism including lipid oxidation were demonstrated in a number of publications [35,36].

We failed to find information about the link of pre-RA stages with oxLDL and AT-oxLDL.

Yet in the experiments. it was demonstrated that oxLDL

1. Disrupted the endothelial barrier, and leaky vessels, in particular the synovial vessel, which does not exclude its entry into the joints with provocation inflammatory reactions [37];
2. Up-regulated maturation of immature dendritic cells, with expression of major histocompatibility complex (MHC) class II and co-stimulatory molecule CD86 [38];
3. Induced long-term proinflammatory cytokine production by monocytes [39].

So. potentially. the increased oxLDL levels at the pre-RA stage are fraught with the provocation of processes that are significant for development of RA.

In eRA. increased levels of oxLDL and AT-oxLDL were demonstrated [40,41,42,43] in correlation with inflammatory markers and complex RA activity indices [44,45]. A very important fact is that elevated levels of oxLDL and AT-oxLDL were independently associated with RA onset [42].

Despite strong evidence linking OS marker levels with ecotoxin exposure, no association between oxidative stress and individual physiological/lifestyle factors was found in our cohorts. This lack of association is due to study limitations. Further clarification of the dose-response effects of tobacco smoking, alcohol consumption. and fish consumption, as well as data on occupational exposure, is needed.

The most significant argument in favor of the provoking influence of ecotoxins and ecotoxin-associated OS on the development of RA is. of course, the link of these factors with RA indices. The only RA index that depended on these factors was the ACPA level. The link was demonstrated in the total FDR cohort as well as at II–IV pre-RA stages and in eRA. It was not detected in the aRA cohort, which, in our opinion, may be due to the diversity of stimuli for the production of these autoantibodies during the full swing of the disease. It is noteworthy that the link between ACCP levels and OS markers was not revealed in SE-carriers, but in non-carriers, this RA index was influenced by the 8-OHdG and AT-oxLDL levels. The intimate relationship between SE and CCP, which determines the production of antibodies to these modified peptides. is well known [46]. It is possible that, in the cohort of SE-carriers, anti-CCP production is stimulated by different factors, as opposed to the less specific production observed in SE non-carriers.

Our data are consistent with the results of a comprehensive analysis of 93 studies, primarily in RA cohorts, which found a link between ACCP (but not RF) positivity. in exposure to sulfur oxide; the complex effects of a combination of ecotoxins on these antibodies were not analyzed [47]. Combined exposure to industrial sulfur dioxide (SO_2_) and nitrogen dioxide (NO_2_) was associated with ACCP positivity [48]. Perhaps the above-mentioned synergistic interaction between the SE and AhR pathways contributes to these links.

The role of OS, including ecotoxin-associated OS in RA pathogenesis, is known [49,50,51,52].

Malondialdehyde-acetaldehyde (MAA), which is formed during the oxidation of membrane lipids, is of particular interest. Its adducts, as immune adjuvants. are due to anti-MAA autoantibodies [53].

MAA adduct formation is increased in RA. Adducts were found in increased quantities in the rheumatoid synovia membrane. and serum anti-MAA antibodies correlated with RA indices [54,55,56].

In RA, correlation of serum ACCP and OS marker protein carbonyl levels was revealed [57]. In RA synovial fluid, ACCP positivity might be associated with oxidant activity (increased malondialdehyde (MDA) and myeloperoxidase (MPO) levels) [58]. In addition, there can be colocalization of citrulline. homocitrulline. and myeloperoxidase in metatarsal synovial tissues of seropositive RA patients [59].

It turned out to be more difficult to trace the molecular mechanisms underlying the link between ACCP production and OS. The process of protein citrullination does not directly involve steps involving reactive oxygen species, lipid peroxides and oxidized lipids. proteins and DNAs [60,61,62]. More than that, in model experiments, ROS-induced PAD (peptidyl arginine deiminase) downregulation was demonstrated [63]. yet this effect appeared to be dose dependent and did not develop when PAD released from stimulated leukocytes was treated with H_2_O_2_ in high concentrations [64]. It was also demonstrated that intracellular ROS may promote intracellular histone H3 citrullination [65].

However, MAA adducts colocalize with citrullinated antigens rheumatoid synovial tissues, and in the modal experiments. it was demonstrated that MAA adduction of citrullinated antigen greatly enhances immune and cellular responses [66]. Dually modified MAA and citrullinated fibrinogen activated macrophage release soluble factors. inducing fibroblast activation and promotion of an aggressive fibroblast phenotype [67].

Thus, it can be assumed that ecotoxin-associated OS might contribute to provokation of ACCP production in RA patients and. importantly. in individuals at preclinical stages of the disease.

## 4. Materials and Methods

### 4.1. Cohorts

Individuals (n = 184) were selected for the cohorts through clinical and laboratory rheumatological examinations of residents of populated areas within the administrative districts of the Republic of Tatarstan.

The nationality of these individuals, determined using information on three generations. was clarified for 163 of them: 55 Russians, 88 Tatars. 10 persons came from mixed Russian–Tatar families, and 30 individuals of other nationalities from the Volga-river region (Udmurts, Mari, Chuvash, Mordvins).

According to the mitochondrial data, the populations of Russians and Tatars living in the Volga-river region have 80% Caucasians and 20% Mongoloids mitotypes [68].

In Russia, Udmurts, Mari, Mordvins and Chuvash belong to the Uralic race (an intermediate position between the Mongoloid and Caucasoid races) [69].

Mitochondrial haplotypes were tested in 69 of the 184 individuals included in this study using HVS1 (hypervariable segment 1) sequencing after PCR and haplogroup determination by online program https://dna.jameslick.com/mthap/ (accessed on 19 March 2026). Most of the determined haplogroups appeared to belong to European (H, V, U, T, n = 64); rare variants (N, Z, C, n = 5) are often found in Mongoloids.

For the purposes of this study, the following cohorts were formed:

Early (e) RA patients (n = 35), with less than 1 year of disease experience (meeting the ACR/EULAR 2010 criteria).

Advanced (a) RA patients (n = 25), with more than 1 year of disease experience.

First-degree relatives of these patients (i.e., kids, siblings, and parents) at pre-RA stages (pre-RA. n = 72).

The Control cohort consisted of 52 healthy individuals without autoimmune and immunoinflammatory diseases in family history, with serum ESR, CRP, RF and ACCP levels within reference values (Controls).

Pre-RA stages were identified according to the recommendations of the Study Group for Risk Factors for Rheumatoid Arthritis (EULAR Standing Committee on Investigative Rheumatology) [70]: 1st—genetic stage (no clinical and laboratory symptoms); 2nd—autoimmune stage (increase levels of ESR and/or CRP. and/or RF. and/or aCCP without any joint symptoms); 3rd—arthralgia (without clinical and detected by instrumental methods signs of arthritis); 4th—undifferentiated arthritis (not meeting the ACR/EULAR 2010 criteria of eRA).

Cohort characteristics are presented in Table 6 and Table 7.

This study was approved by the Ethical Committee of the Kazan State Medical Academy. Kazan. Russia (Permit nr 15/1/2002). The written consent to conduct studies and to allow publication of the results was received from all the individuals involved in this study according to the legal requirements in Russia. The approval was confirmed on 16 February 2023 (nr 5/02).

### 4.2. Clinical and Laboratory Examination

The clinical and laboratory RA indices recommended by EULAR and Russian clinical recommendations were included in the analysis.

Laboratory RA indices in all the cohorts included Westergren erythrocyte sedimentation rate (ESR), C-reactive protein (CRP), rheumatoid factor (RF) and antibodies to cyclic citrullinated peptides (aCCP).

### 4.3. Laboratory Tests

Oxidative stress markers were tested using the following kits: serum levels of oxidized proteins (AOPP)—AOPP kit (nr KR7811W. Immun diagnostik AG, Bensheim, Germany); indicator of oxidative damage to intracellular DNA (serum levels of oxidized guanin (8-OHdG))—HT8-oxo-dG ELISA Kit II (nr 4380-192-K. Trevigen. Inc., Gaithersburg, MD, USA); serum levels of low density lipoproteins (oxLDL)—Mercodia Oxidized LDL ELISA (nr 10-1143-01. Mercodia AB, Uppsala, Sweden); antibodies to oxLDL—IMTEC-oxLDL-Antibodies (nr ITC59500. HUMAN GmbH, Wiesbaden, Germany).

Mercodia Oxidized LDL ELISA is a unique sandwich ELISA based on the proprietary mouse monoclonal antibody 4E6. which is directed against a conformational epitope in oxidized ApoB-100—two monoclonal antibodies are directed against separate antigenic determinants on the oxidized apolipoprotein B molecule.

Spectral characteristics of AOPP correspond to several chromophores, which include dityrosine, carbonyls and pentosidine and AOPP results from oxidative stress [71,72].

Serum C-Reactive Protein (CRP)—High Sensitivity C-Reactive Protein (hs-CRP) ELISA Kit (Catalog number 80955. Crystal Chem, Downers Grove, IL, USA).

Serum antibodies to cyclic citrullinated peptides (aCCP)—Human ACPA ELISA Kit. ARIGO (nr ARG80351, Taiwan, China).

Serum rheumatoid factor (RF)—Human Rheumatoid Factor IgM ELISA Kit. ARIGO (nr ARG80411, Taiwan, China).

### 4.4. HLA Typing

HLA typing was performed by DNA sequencing of the DRB1 gene using the HLA holotype kit (Omixon, Budapest, Hungary). Briefly, the isolated DNA concentration was estimated using an Implen NP80 NanoPhotomer (Fisher Scientific, Pittsburgh, PA, USA) and long-range PCR was performed. Then, amplicon pools were fragmented, ends were repaired, and barcodes were ligated. After individual sample barcoding, they were equimolarly pooled and sequenced using a Miseq instrument (Illumina, San Diego, CA, USA) in the pair-end reading mode with read length of 251bp. Resulting reads were analyzed using the HLA Twin v.4.9.0 software (Omixon, Hungary) with IPD-IMGT/HLA Database version 3.56.0. with two algorithms, including de novo assembly and mapping against the IPD-IMGT/HLA database [73].

### 4.5. Phisiological and Lifestyle-Related Factors

The following information was collected during the rheumatological examination:

1. Age. years;
2. Body mass index;
  Height and weight measurements were made; body mass index (BMI) was calculated using an online calculator.
3. Education level;
  Education was dichotomized into low educational level (secondary and high school graduates) and high educational level (university graduates) [74].
4. Fish consumption. yes or no;
5. Coffee consumption. cups/day;
6. Alcohol consumption. no or rarely or regularly;
7. Active tobacco smoker for the last year. yes or no;
8. Passive tobacco smoker for the last year. yes or no;
9. Number of childbirths (women).

### 4.6. Ecological Analysis

Information was collected on the parameters of the environmental situation in the administrative regions of the Tatarstan Republic in 2008–2018 (according to publicly available reports from the Ministry of Ecology of Republic of Tatarstan). We used average values over 10 years for each parameter. In particular, factors such as the content of carbon monoxide, hydrocarbons (without VOCs), solid particles, VOCs, sulfur dioxide and nitrogen oxide in the atmospheric air were considered [75].

Soil sampling for analysis in the settlements of the persons included in our cohorts was carried out in the areas not influenced by agriculture and industry. The mixed soil sampling was carried out by an Eidelman soil drill. Representative soil samples taken for analysis (lower layer soil (A1) (5–25 cm). upper layer soil (Ad) (0–5 cm)) were dried in a well-ventilated room.

The total content of elements of different hazard classes was determined in the soil samples: Al. As. Cd. Pb. Sr. Cu. Fe. Mn. Mo. Ni. Co. Cr. Se and Zn [76].

### 4.7. Statistical Analysis

1. Mann–Whitney U test [77].
2. Spearman rank order correlation [78,79].
3. Multiple regression analysis.
4. Chi-square.

While commenting the results of statistical analysis, we made a distinction between “presumable significance” (*p*-value < 0.05 and >0.01) and “reliable significance” (*p*-value < 0.01).

## 5. Limitations of This Research

We limited our studies to linking ecotoxin-induced oxidative stress and RA indices in RA and in the FDR cohort at pre-RA stages. However, these results need to be supplemented with studies of the possible activation of signaling pathways important for disease pathogenesis by environmental factors. It might be necessary to evaluate the indirect effects of ecotoxins through the possible enhancement of the infectious syndrome in the pre-RA and eRA stages.

## Figures and Tables

**Table 1:** Serum levels of OS markers in the eRA, aRA, pre-RA and Control cohorts.

Mann–Whitney U Test (w/Continuity Correction) by Variable Group. Marked Tests Are Significant at *p* < 0.05000										
Variable	Rank Sum FDR	Rank Sum Controls	U	Z	*p*-Value	Z	*p*-Value	Valid N	Valid N	2*1sided
8-OHdG	3319	2037	1217	0.28758	0.773666	0.28761	0.773642	63	40	0.774673
**oxLDL**	**3964.5**	**1706.5**	**845.5**	**3.15597**	**0.0016**	**3.15599**	**0.0016**	**65**	**41**	**0.001399**
AT-oxLDL	3025	2646	1072	−1.86902	0.06162	−1.86904	**0.061618**	62	44	0.061427
AOPP	3404.5	2160.5	1214.5	0.76893	0.441932	0.769	0.441893	62	43	0.441657
variable	**eRA**	**Controls**	U	Z	*p*-value	Z	*p*-value	Valid N	Valid N	2*1sided
8-OHdG	1309.5	1465.5	645.5	0.368782	0.712291	0.368806	0.712272	34	40	0.709893
**oxLDL**	**1273**	**1283**	**422**	**2.240797**	**0.02504**	**2.240835**	**0.025037**	**30**	**41**	**0.024375**
AT-oxLDL	931	1625	553	−0.479702	0.63144	−0.47971	0.631434	27	44	0.633619
AOPP	1274	1427	481	1.833149	0.066781	1.833276	0.066762	30	43	0.066563
variable	**aRA**	**Controls**	U	Z	*p*-value	Z	*p*-value	Valid N	Valid N	2*1sided
8-OHdG	586.5	1304.5	355.5	−0.971483	0.331309	−0.971547	0.331277	21	40	0.330379
oxLDL	608	1283	398	−0.176679	0.85976	−0.176686	0.859755	20	41	0.860965
AT-oxLDL	863	1348	358	1.707101	0.087804	1.707101	0.087804	22	44	0.087879
AOPP	773	1307	361	1.286864	0.198143	1.28707	0.198071	21	43	0.199855
variable	**FDR**	**eRA**	U	Z	*p*-value	Z	*p*-value	Valid N	Valid N	2*1sided
8-OHdG	3060	1693	1044	−0.20036	0.841198	−0.20039	0.841177	63	34	0.842014
oxLDL	3213	1347	882	0.74059	0.458941	0.7406	0.458935	65	30	0.461066
AT-oxLDL	2685.5	1319.5	732.5	−0.92816	0.353323	−0.92817	0.353319	62	27	0.353195
AOPP	2722	1556	769	−1.3368	0.181288	−1.33689	0.181259	62	30	0.18233
variable	**FDR**	**aRA**	U	Z	*p*-value	Z	*p*-value	Valid N	Valid N	2*1sided
8-OHdG	3352	1401	651	−1.87684	0.060541	−1.87699	0.060521	73	24	0.060228
oxLDL	3156	1309	600	−1.89972	0.057471	−1.89975	0.057467	71	23	0.057102
AT-oxLDL	3375	1090	747	−0.39738	0.691084	−0.39739	0.691083	72	22	0.693127
AOPP	3166	929	698	0.24803	0.804109	0.24806	0.804090	69	21	0.805556
variable	**aRA**	**eRA**	U	Z	*p*-value	Z	*p*-value	Valid N	Valid N	2*1sided
8-OHdG	509.5	1030.5	278.5	−1.35127	0.17661	−1.35142	0.176563	21	34	0.175357
oxLDL	416	859	206	−1.85158	0.064088	−1.85184	0.064049	20	30	0.063708
**AT-oxLDL**	**662.5**	**562.5**	**184.5**	**2.25128**	**0.024368**	**2.25134**	**0.024365**	**22**	**27**	**0.022769**
AOPP	520.5	805.5	289.5	−0.47847	0.632313	−0.47852	0.632282	21	30	0.628485

**Table 2:** The link of serum levels of OS markers and AT-oxLDL in cohorts.

Cohort	Pair of Variables	Valid N	Spearman R	t (N − 2)	*p*-Value
Control	8-OHdG and AT-oxLDL	38	−0.507879	−3.53747	0.001134
	oxLDL and AT-oxLDL	41	−0.302626	−1.98288	**0.054456**
	AOPP and AT-oxLDL	35	0.577613	4.06479	0.000280
FDR	8-OHdG and AT-oxLDL	58	−0.353567	−2.82855	0.006477
	oxLDL and AT-oxLDL	62	−0.580879	−5.52767	0.000001
	AOPP and AT-oxLDL	56	0.666826	6.57550	0.000000
eRA	8-OHdG and AT-oxLDL	26	−0.220588	−1.10795	0.278864
	oxLDL and AT-oxLDL	25	−0.537796	−3.05925	0.005558
	AOPP and AT-oxLDL	24	0.609395	3.60504	0.001572
aRA	AT-oxLDL and 8-OHdG	18	−0.097057	−0.390071	0.701628
	AT-oxLDL and oxLDL	18	−0.075335	−0.302200	0.766392
	AT-oxLDL and AOPP	18	0.446852	1.997982	0.063010

Spearman rank order correlations MD pairwise deleted. Marked correlations are significant at *p* < 0.05000.

**Table 3:** Age and body mass index in high and low educational level cohorts.

Education Level	Low (n = 123)	High (n = 53)
Age, years	55.00 (61)	41.00 (77)
MW, *p*	0.000009	
BMI *	28.54 (24)	24.27 (34)
MW, *p*	0.000354	

* Body mass index. Median (IQR) is presented.

**Table 4:** The link between BMI and age with OS markers level.

Pair of Variables	Valid	Spearman	t (N − 2)	*p*-Value
Age and BMI	165	0.413281	5.79441	0.000000
BMI and 8-OHdG	153	0.104471	1.29083	0.198736
BMI and oxLDL	150	0.235871	2.95281	0.003664
BMI and AT-oxLDL	148	−0.259878	−3.25184	0.001424
Age and 8-OHdG	162	0.000525	0.00664	0.994706
Age and oxLDL	158	0.177208	2.24893	0.025917
Age and AT-oxLDL	157	−0.250577	−3.22247	0.001549

Spearman rank order correlations (ADDITIONAL STATISTIC); MD pairwise deleted; Marked correlations are significant at *p* < 0.05000.

**Table 5:** The frequency of tobacco smoking and alcohol. fish and coffee consumption in lower and higher educational cohorts (%).

Educational Level	Active or Passive Smoker No/Yes (%)	Alcohol No/Rarely (%)	Fish No/Yes (%)	Coffee No/Yes (%)
Lower	81/48 (37.2)	64/65 (50.4)	25/103 (80.5)	68/60 (46.9)
Higher	42/11 (20.8)	18/35 (67.3)	17/37 (68.5)	19/35 (64.8)
Chi-square. *p*	0.032	0.054	0.081	0.027

**Table 6:** Characteristics of the cohorts.

Cohorts	Controls	Pre-RA-FDR	eRA	aRA
Age. years. median (IQR *)	51.0 (51.0)	55.0 (55.0)	56.0 (56.0)	51.0 (51.0)
Gender female/male (female %)	49/3 (94.2)	65/7 (90.3)	34/1 (97.1)	25/25 (100.0)
BMI. median (IQR *)	30.5 (18.25)	27.5 (18.25)	28.5 (20.75)	29.0 (39.0)
RA experience. years. median (IQR *)			0.57 (2.0)	6.9 (7.0)
DAS28-ESR score. median (IQR *). low/moderate/high activity (%)			3.97 (54.0) 28.6/8.6/62.8	3.4 (40.0) 13.8/40.4/45.8
HAQ score. median (IQR *)			1.25 (38.0)	0.3 (8.5)
ESR. mm/hour. median (IQR *)			33.0 (32.0)	30.0 (27.0)
CRP. mg/mL. median (IQR*)			5.5 (10.0)	7.3 (45.0)
RF. IU/mL. median (IQR *)			21.45 (38.0)	22.0 (45.5)
RF pos (%)			62.5	48.0
aCCP. U/mL. median (IQR *)			38.24 (19.0)	17.7 (67.0)
aCCP pos (%)			80.0	68.0

* IQR—interquartile range.

**Table 7:** Characteristics of cohorts of FDR at I–IV pre-clinic RA stages.

preRA Stage	I (n = 11)	II (n = 17)	III (n = 32)	IV (n = 19)
Age. years. median (IQR *)	36.55 (19)	52.00 (19.5)	52.50 (19)	51.00 (14)
Gender female/male (female %)	11/0 (100)	16/1 (94.1)	31/1 (96.9)	18/1 (94.7)
BMI	24.22 (11)	29.08 (25)	25.46 (9)	28.82 (23)
HAQ score. median (IQR *)	×	×	0.50 (17)	0.63 (75)
ESR. mm/hour. median (IQR *)	12.00 (13)	35.00 (22.5)	13.50 (14)	23.50 (27)
CRP. mg/mL. median (IQR *)	0.77 (7)	3.50 (2)	2.33 (8)	3.50 (2.5)
RF. IU/mL. median (IQR *)	9.00 (3)	13.00 (54)	11.00 (30)	7.02 (120)
RF pos (%)	0	29.4	34.5	17.6
aCCP. U/mL. median (IQR *)	9.40 (3)	23.65 (47)	11.54 (59)	9.34 (38.5)
aCCP pos (%)	0	63.6	33.33	31.25

* IQR—interquartile range.

## Data Availability

The original contributions presented in this study are included in the article/Supplementary Materials; further inquiries can be directed to the corresponding author.

## Supplementary Materials

The following supporting information can be downloaded at: https://www.mdpi.com/article/10.3390/ijms27073328/s1.
